# Supplementary material for: Embedding oxophilic rare-earth single atom in platinum nanoclusters for efficient hydrogen electro-oxidation
Source: Nat Commun. 2023 Jun 24;14:3767. doi: 10.1038/s41467-023-39475-5 (PMC10290706; doi:10.1038/s41467-023-39475-5)
Supplement: Supplementary file 1 — Supplementary Information [file 41467_2023_39475_MOESM1_ESM.pdf]

Supplementary Information for

Embedding oxophilic rare-earth single atom in platinum nanoclusters for efficient hydrogen electro-oxidation

Wang et al.

Supplementary Figures

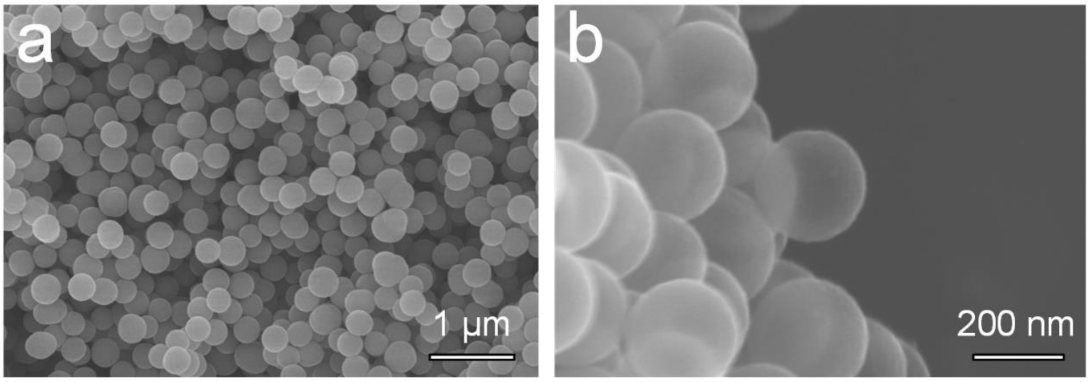

Supplementary Fig. 1 SEM images of HCS material. a, Low magnification image. **b,** High magnification image.

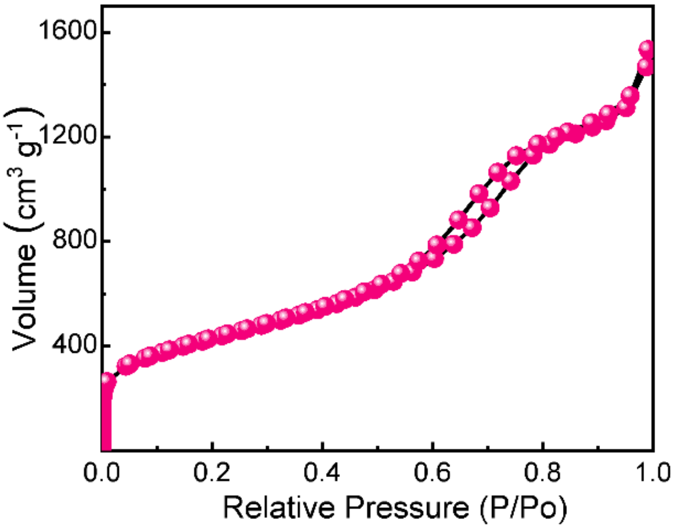

Supplementary Fig. 2 N_2 adsorption/desorption isotherm of HCS material.

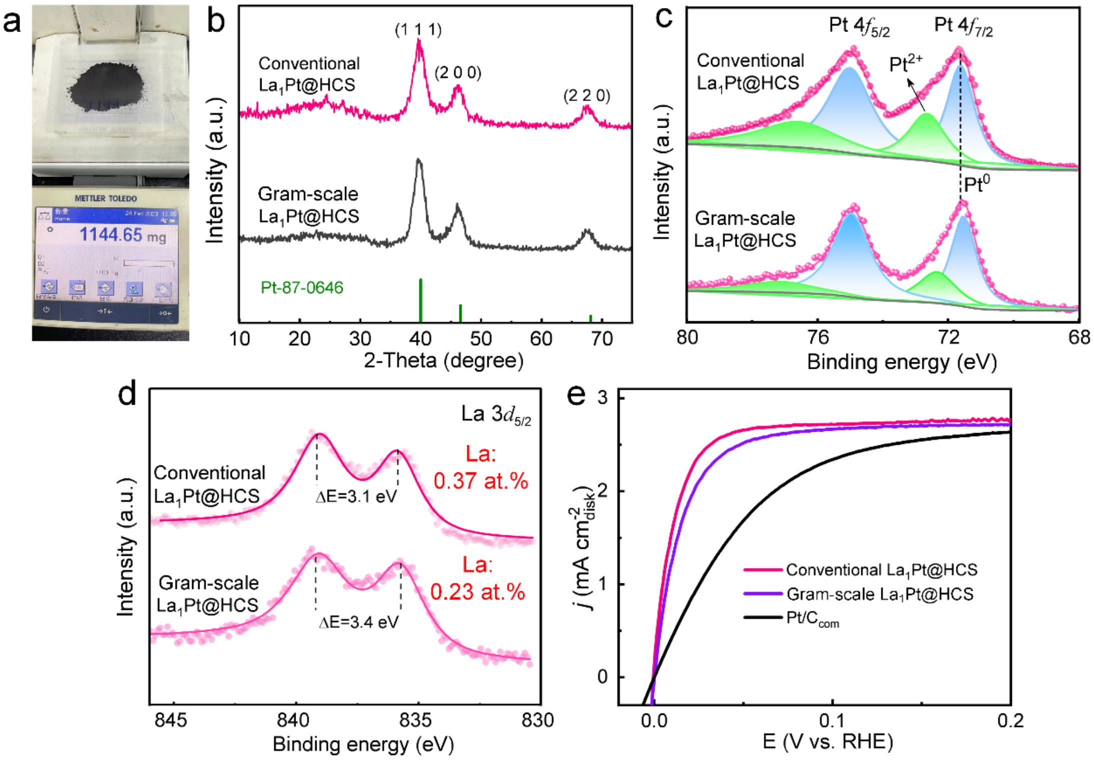

Supplementary Fig. 3 Structural and HOR performance characterization of conventional La_1Pt@HCS and the one by gram-scale production. a, Digital photo of gram-scale La_1Pt@HCS. **b,** XRD. **c,** Pt 4*f* XPS spectra. **d,** La 3*d* XPS spectra. **e,** HOR polarization curves in H_2-saturated 0.1 M KOH with a scan rate of 5 mV s^{-1} at a rotating rate of 1600 rpm. The gram-scale synthesis of La_1Pt@HCS was realized by increasing the dosage of HCS from 35 mg to 1000 mg in a doubled-volume ampoule with the proportional feeding of $Pt(acac)_2$ and $La(acac)_3$, which is the maximal production capability in our lab due to the equipment limitations.

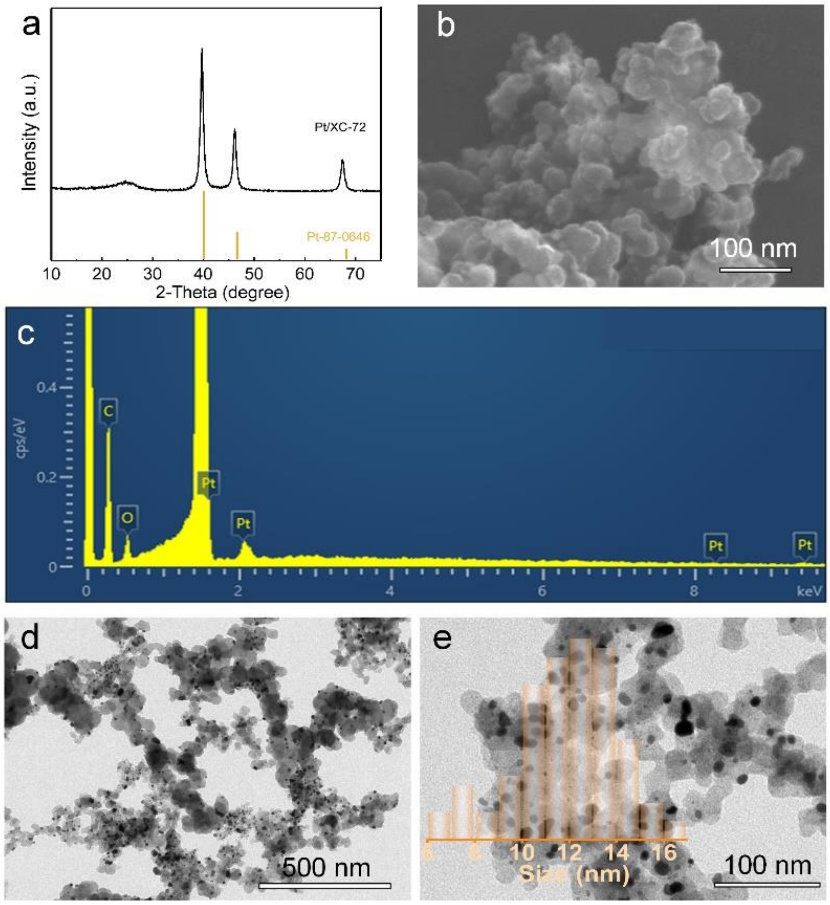

Supplementary Fig. 4 Structural characterization of XC-72 carbon-supported Pt particles (Pt/XC-72 for short). a, XRD pattern. **b**, SEM image. **c**, EDX spectrum. **d**, **e**, TEM images (inset: size distribution histogram of Pt particles).

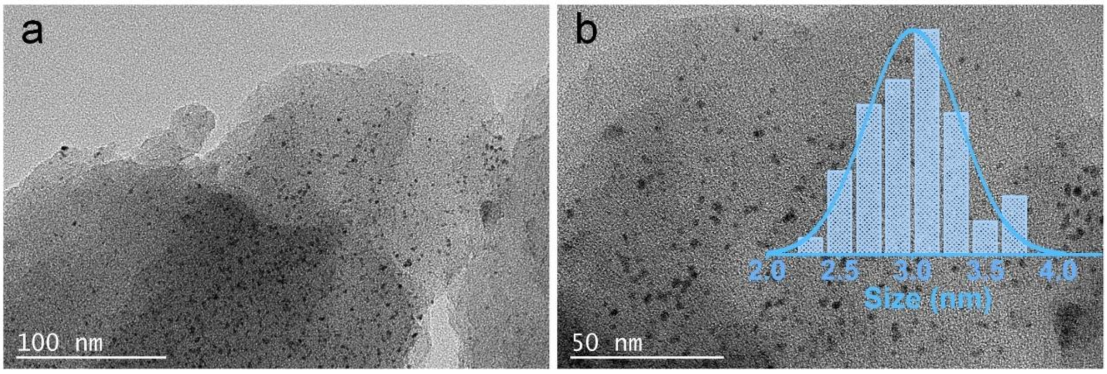

Supplementary Fig. 5 TEM images of La_1Pt NCs inside PAC support. a, Low magnification image. **b,** High magnification image (inset: size distribution histogram of La_1Pt NCs).

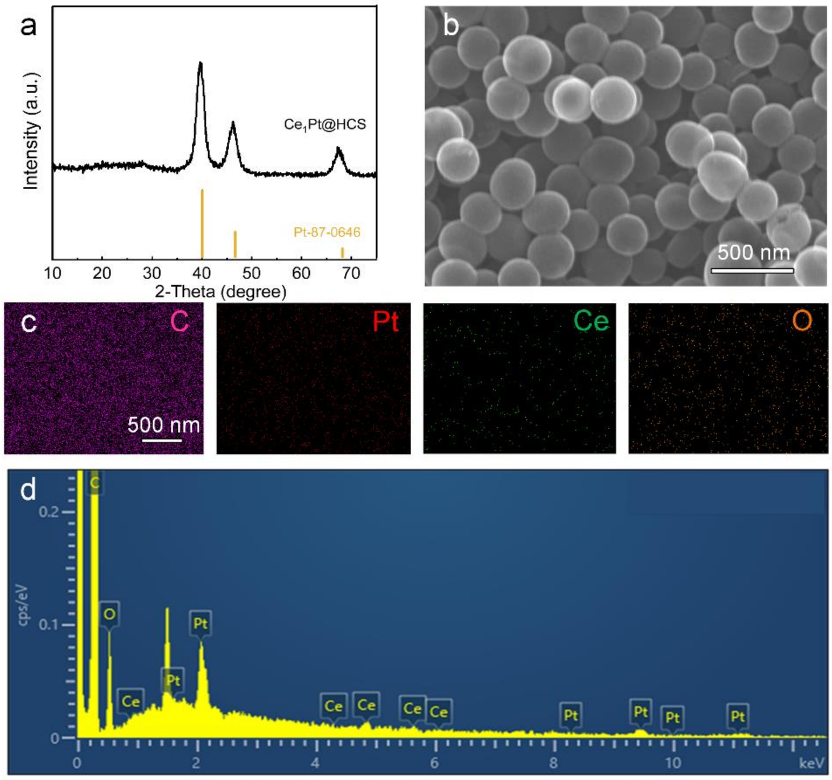

Supplementary Fig. 6 Structural characterizations of Ce_1Pt@HCS. a, XRD pattern. **b**, SEM image. **c**, EDX mapping images of **b**. **d**, EDX spectrum.

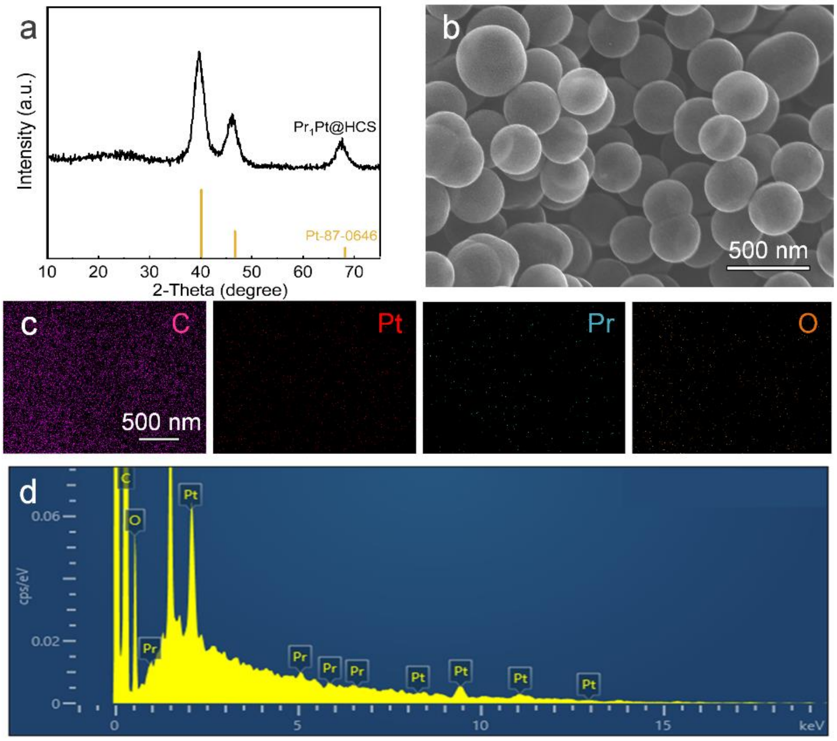

Supplementary Fig. 7 Structural characterizations of Pr_1Pt@HCS. a, XRD pattern. **b**, SEM image. **c**, EDX mapping images of **b**. **d**, EDX spectrum.

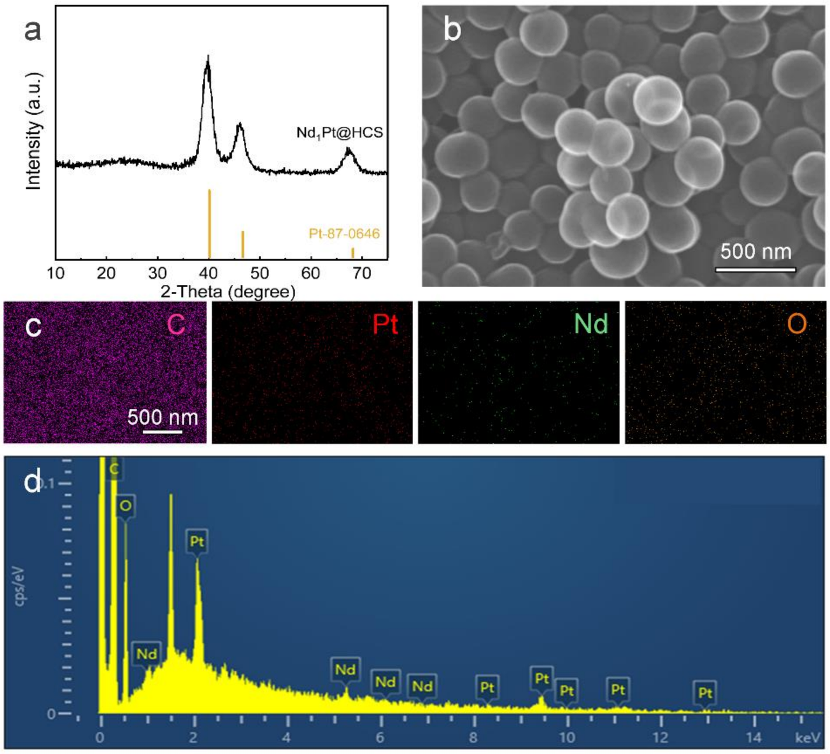

Supplementary Fig. 8 Structural characterizations of $Nd_1Pt@HCS$. a, XRD pattern. **b**, SEM image. **c**, EDX mapping images of **b**. **d**, EDX spectrum.

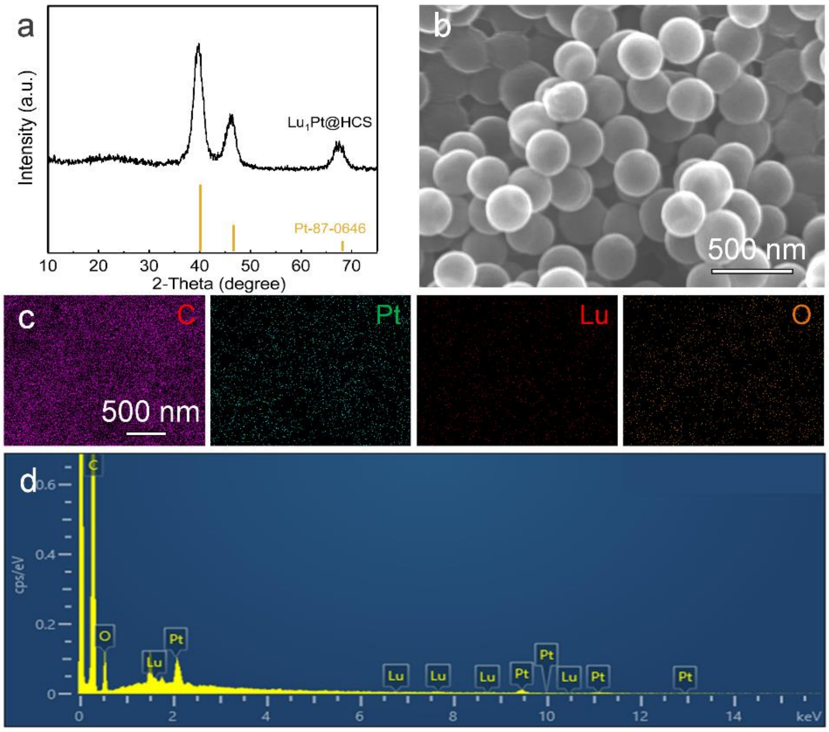

Supplementary Fig. 9 Structural characterizations of $Lu_1Pt@HCS$. a, XRD pattern. **b**, SEM image. **c**, EDX mapping images of **b**. **d**, EDX spectrum.

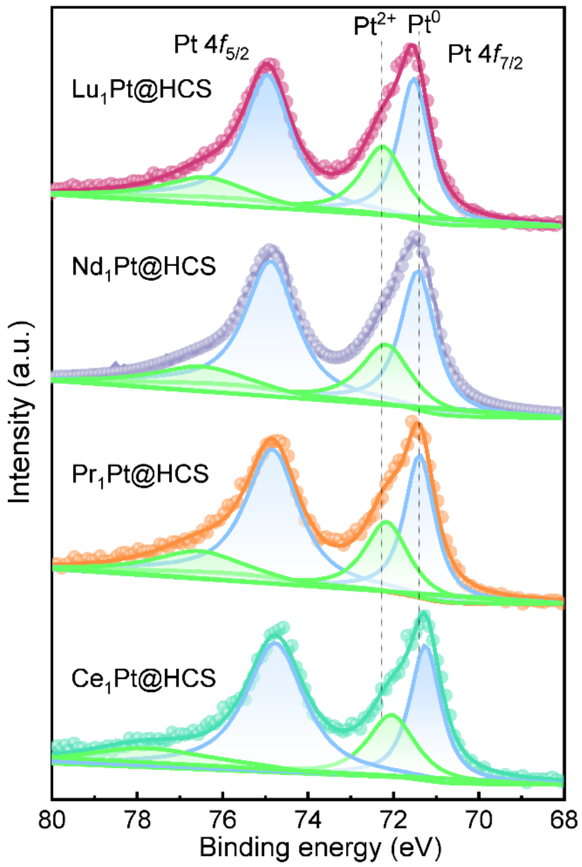

Supplementary Fig. 10 Pt 4*f* XPS spectra comparison of Ln_1Pt@HCS.

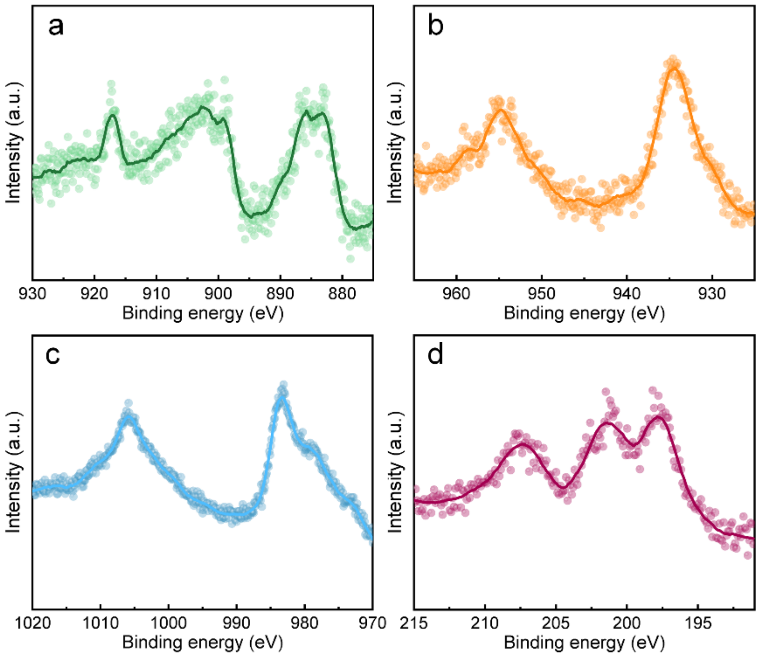

Supplementary Fig. 11 High-resolution XPS spectra of Ln_1Pt@HCS. a, Ce 3*d*; **b**, Pr 3*d*; **c**, Nd 3*d*; **d**, Lu 4*d*.

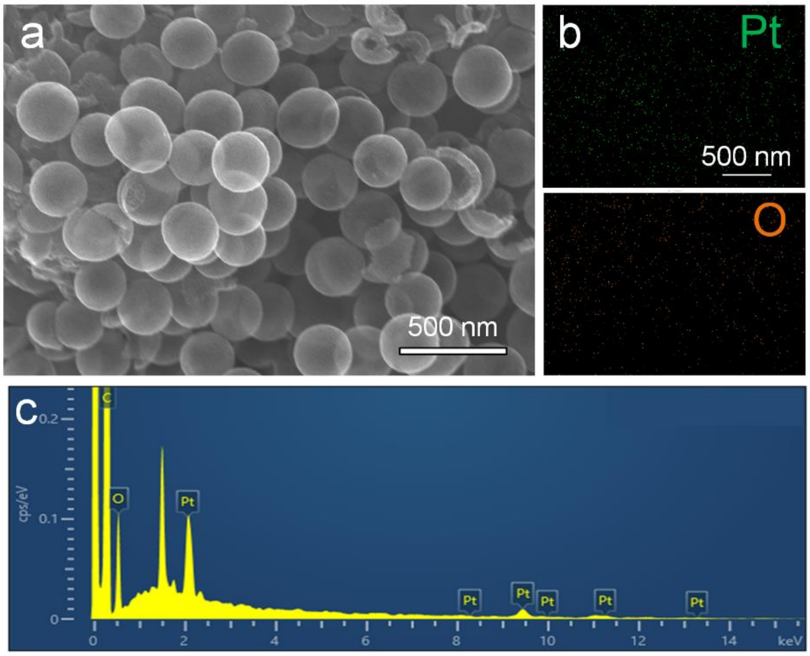

Supplementary Fig. 12 Structural characterizations of Pt@HCS. a, SEM images. **b**, EDX mapping images of **a**. **c**, EDX spectrum.

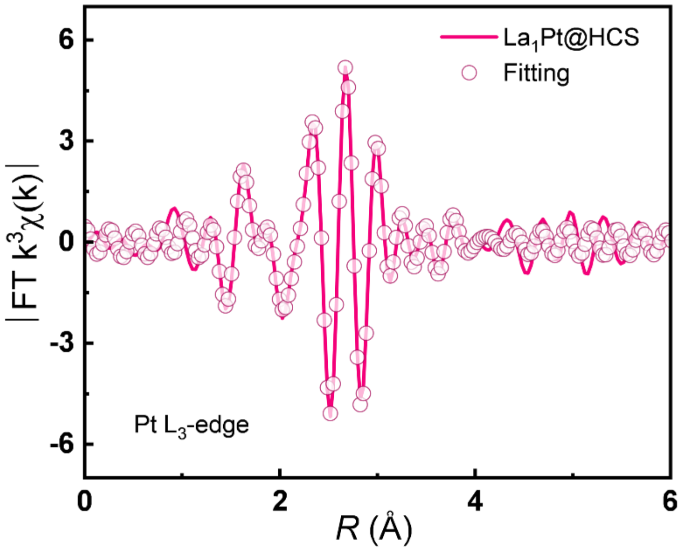

Supplementary Fig. 13 FT k^3-weighted EXAFS experimental and fitting spectrum of La_1Pt@HCS in R real space at Pt L_3-edge.

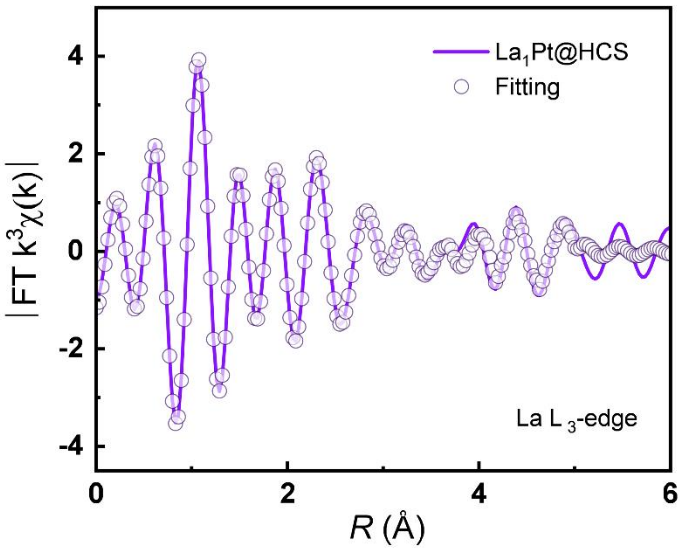

Supplementary Fig. 14 FT *k*^{3}-weighted EXAFS experimental and fitting spectrum of La$_1$Pt@HCS in *R* real space at La L$_3$-edge.

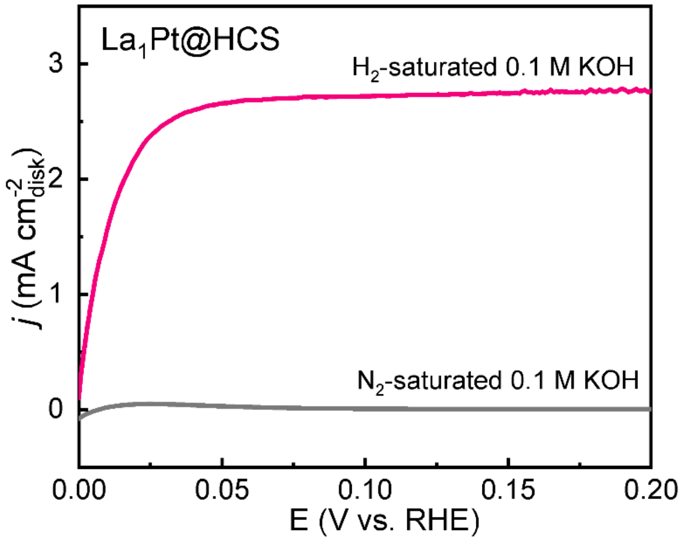

Supplementary Fig. 15 Comparison of activity under different saturated gases. Polarization curves of La$_1$Pt@HCS in H_2 and N_2-saturated 0.1 M KOH at a scan rate of 5 mV s^{-1} and a rotating speed of 1600 rpm.

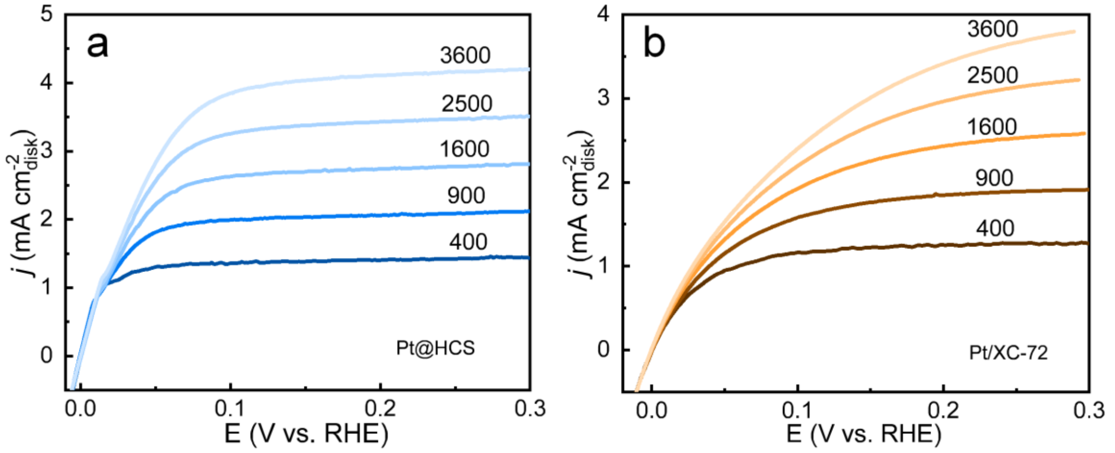

Supplementary Fig. 16 Polarization curves at different rotation rates. a, Pt@HCS; **b**, Pt/XC-72.

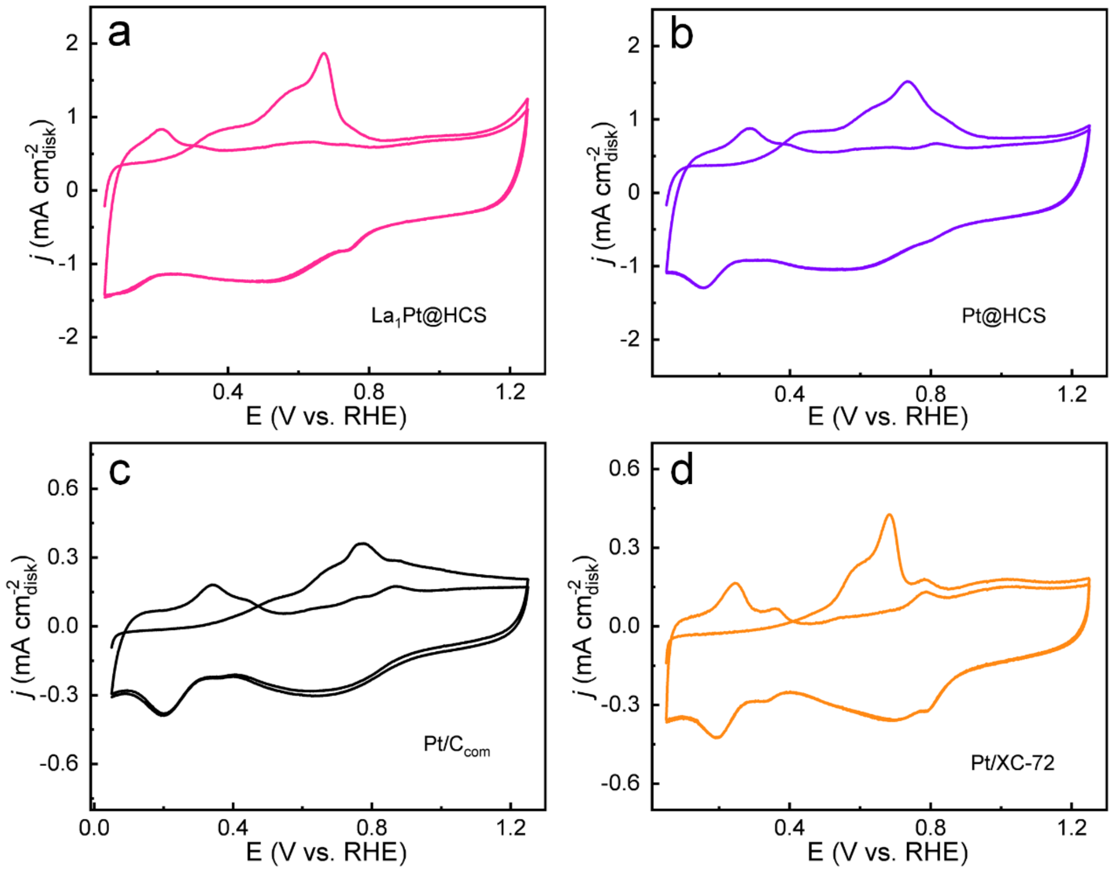

Supplementary Fig. 17 CO-stripping curves in 0.1 M KOH solution at a scan rate of 50 mV s^{-1}. a, La_1Pt@HCS; **b**, Pt@HCS; **c**, Pt/C_{com}; **d**, Pt/XC-72.

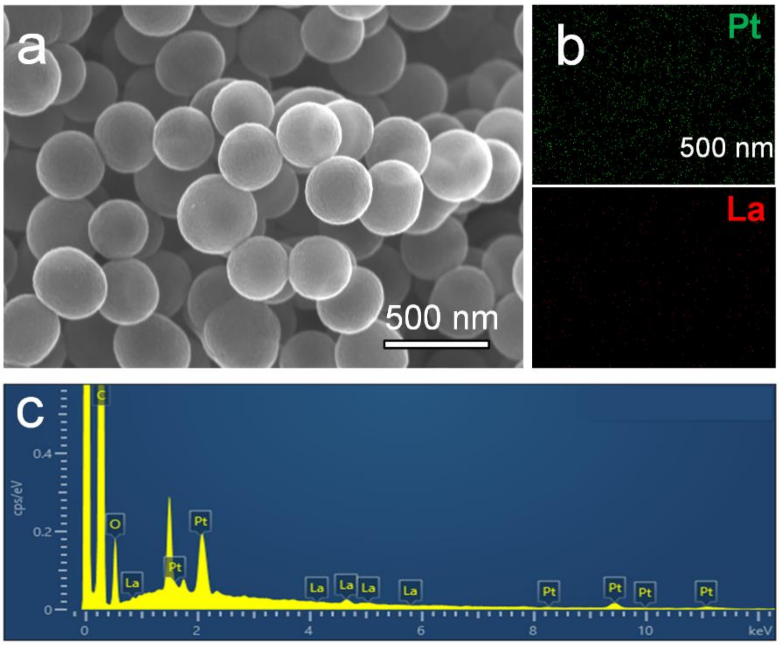

Supplementary Fig. 18 The morphology and composition of La$_1$Pt@HCS after ADT. a, SEM images. **b**, Elemental mappings of **a**. **c**, EDX spectrum.

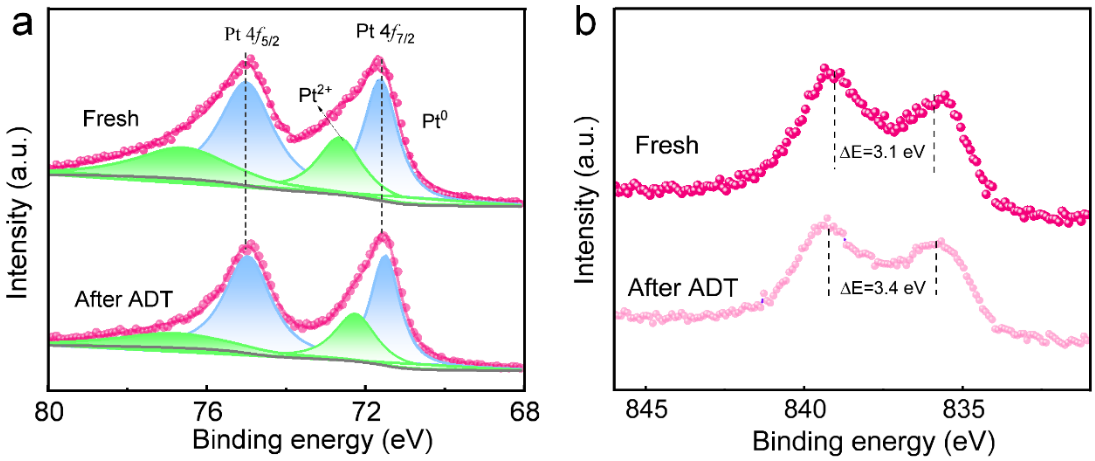

Supplementary Fig. 19 XPS of La$_1$Pt@HCS before and after ADT. a, High-resolution XPS Pt 4*f* spectra. **b**, High-resolution XPS La 3$d_{5/2}$ spectra.

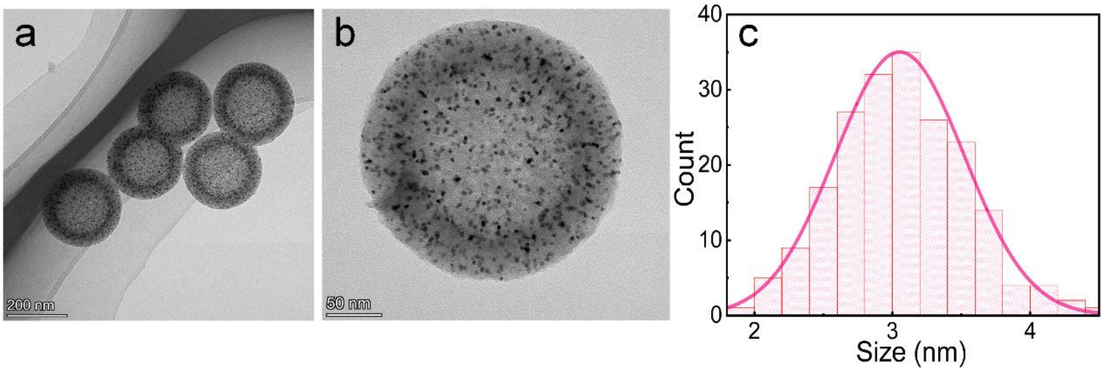

Supplementary Fig. 20 Morphological characterization of La_1Pt@HCS after ADT. **a**, **b**, TEM. **c**, The size distribution histograms of Pt NCs (Number of samples: 200).

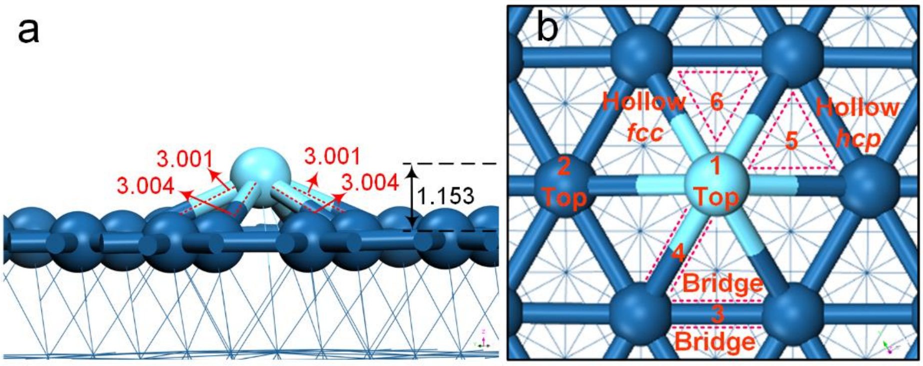

Supplementary Fig. 21 Optimized La_1Pt NCs model. **a**, Stable configuration of La_1Pt. Unit of bond length: Å. **b**, Possible adsorption sites for reaction intermediates. Pt, dark blue; La, dark cyan.

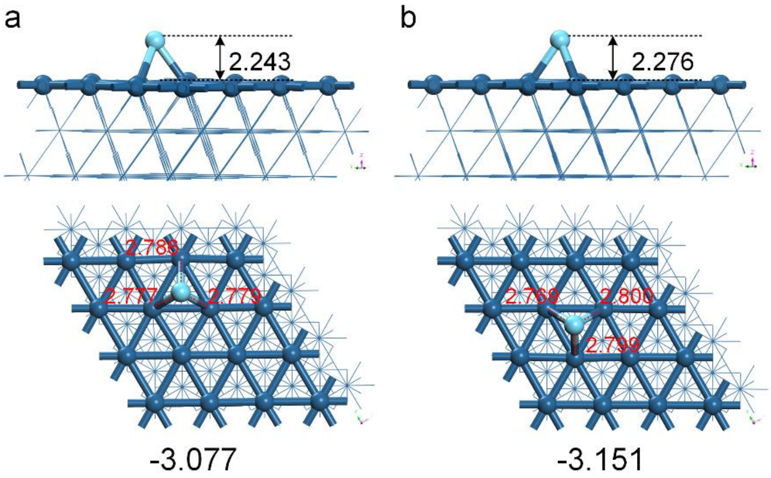

Supplementary Fig. 22 Models of adsorbed La_1 on Pt(111). a, adsorbed La at the hcp site. **b,** adsorbed La at the fcc site. Unit of bond length: Å. Pt, dark blue; La, dark cyan. Bottom values represent formation energies (Unit: eV).

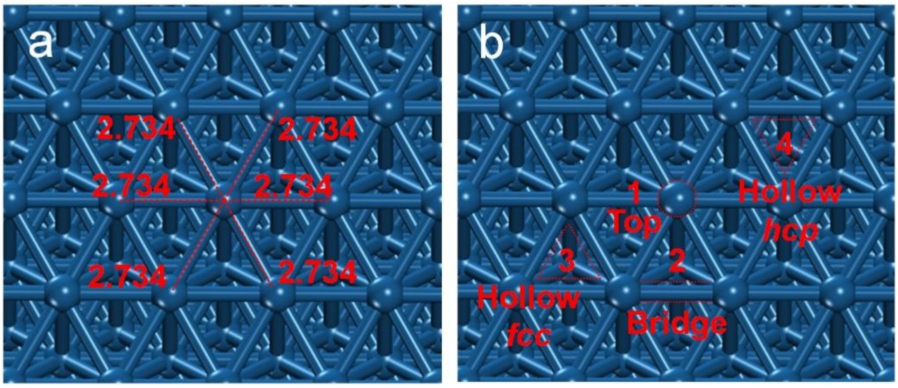

Supplementary Fig. 23 The constructed P(111) model. a, Stable configuration. Unit of bond length: Å. **b**, Possible adsorption sites for reaction intermediates. Pt, dark blue.

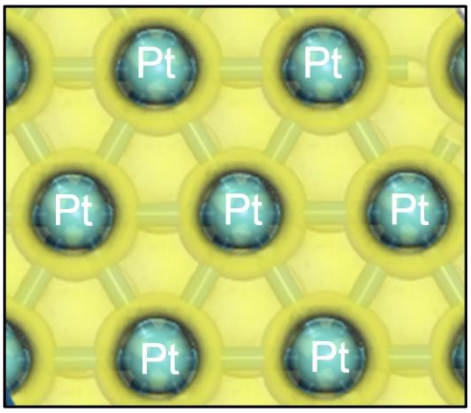

Supplementary Fig. 24 Differential charge densities of Pt NCs. Yellow, electron accumulation; cyan, electron depletion.

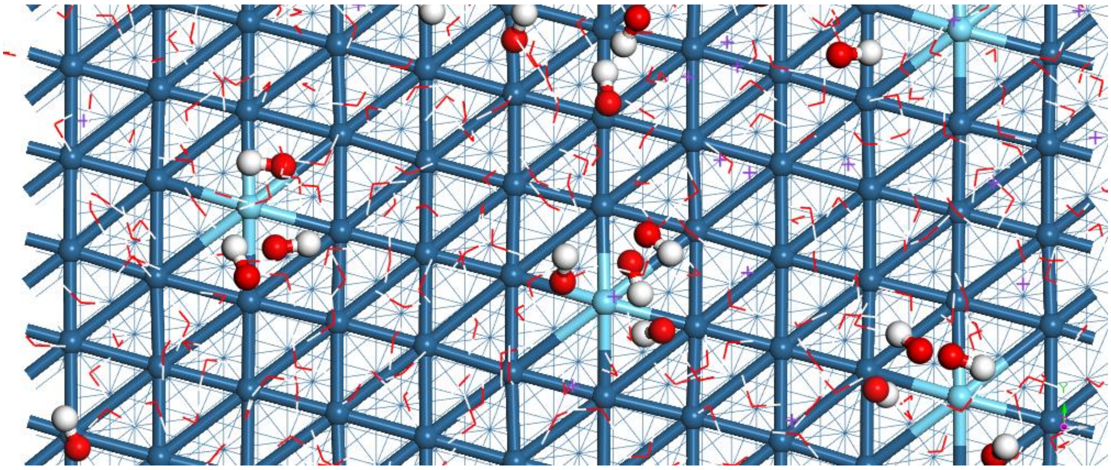

Supplementary Fig. 25 A part of snapshots for the dynamic process of OH^- adsorption on La_1Pt NCs. Pt, dark blue; La, dark cyan. O, red; H, white.

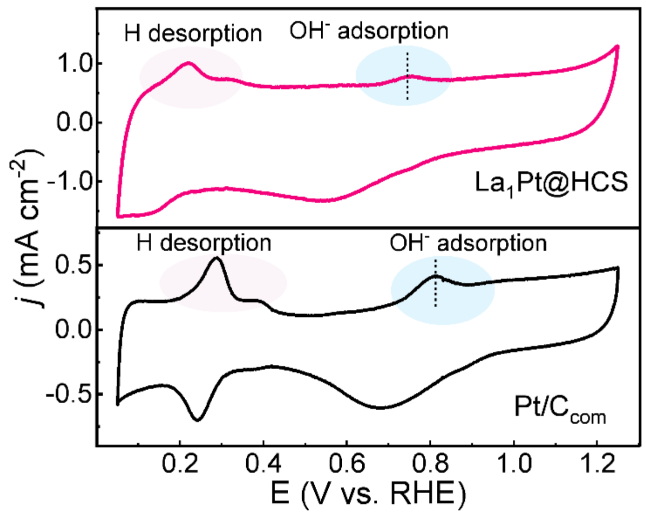

Supplementary Fig. 26 CV curves of La_1Pt@HCS and Pt/C_{com} in Ar-saturated 0.1 M KOH solution.

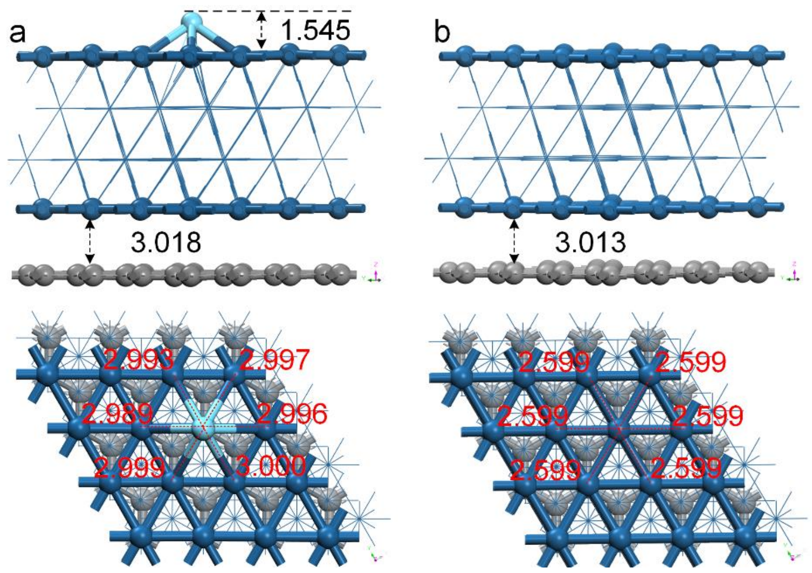

Supplementary Fig. 27 Optimized models. a, La_1Pt NCs@C; **b,** Pt NCs@C.

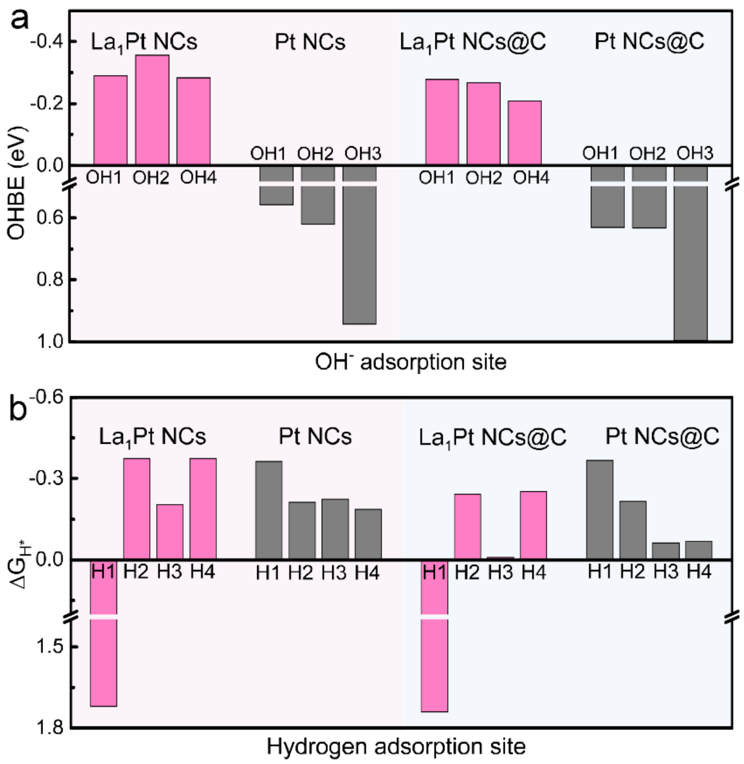

Supplementary Fig. 28 Binding strength of HOR intermediates for La_1Pt and Pt with or without carbon support. a, OHBE; **b,** ΔG_{*H}.

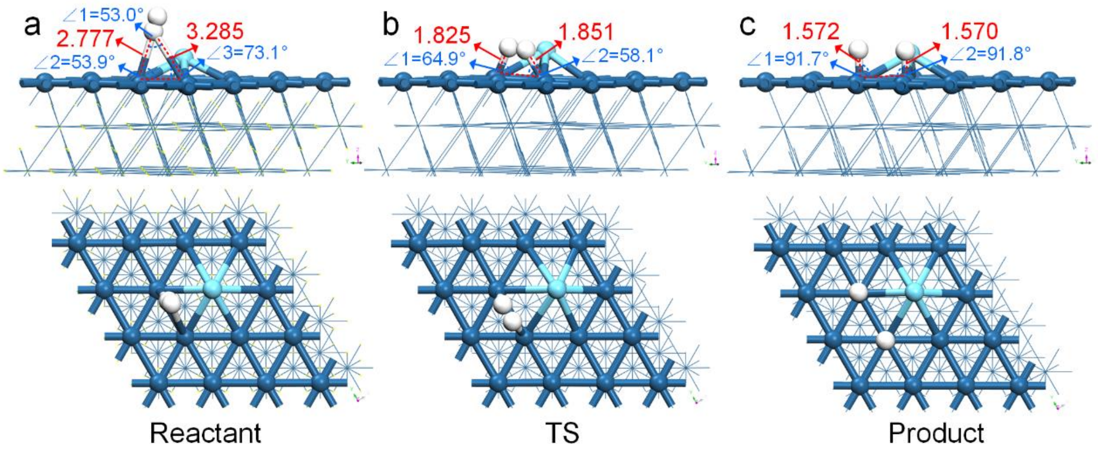

Supplementary Fig. 29 The Tafel reaction on La_1Pt NCs models. a, Reactant; **b**, transition state (TS); **c**, Product. Unit of bond length: Å. Pt, dark blue; La, dark cyan; H, white.

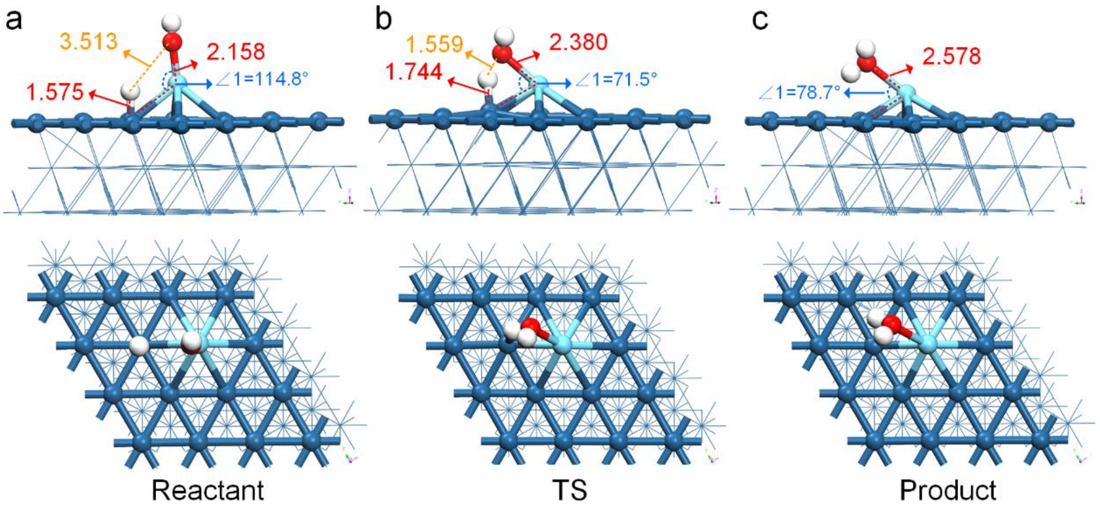

Supplementary Fig. 30 The water formation reaction on La$_1$Pt NCs models. a, Reactant; **b**, TS; **c**, Product. Unit of bond length: Å. Pt, dark blue; La, dark cyan; O, red; H, white.

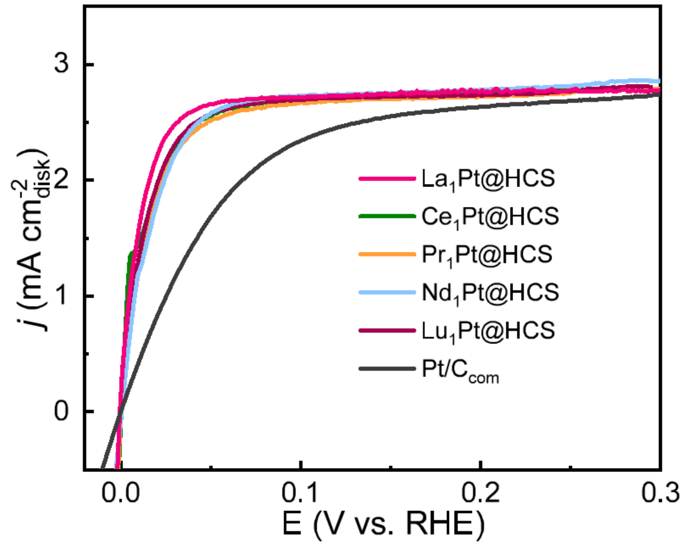

Supplementary Fig. 31 Electrocatalytic performance. HOR polarization curves on La$_1$Pt@HCS, Ce$_1$Pt@HCS, Pr$_1$Pt@HCS, Nd$_1$Pt@HCS, Lu$_1$Pt@HCS, and Pt/C$_{com}$ in H_2-saturated 0.1 M KOH at a rotation rate of 1600 rpm with a scan rate of 5 mV s^{-1}.

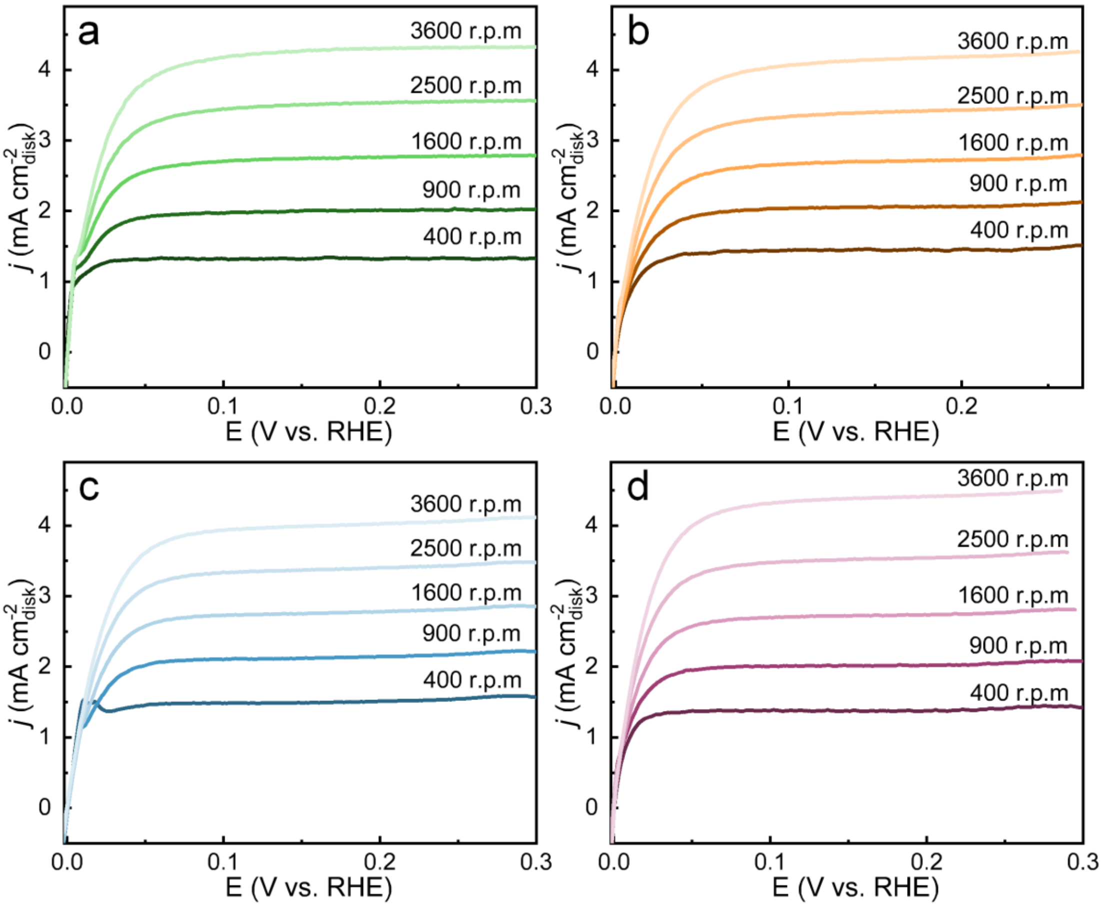

Supplementary Fig. 32 Polarization curves at varied speeds in H_2-saturated 0.1 M KOH at a rotation rate of 1600 rpm with a scan rate of 5 mV s^{-1}. a, Ce_1Pt@HCS; **b**, Pr_1Pt@HCS; **c**, Nd_1Pt@HCS; **d**, Lu_1Pt@HCS.

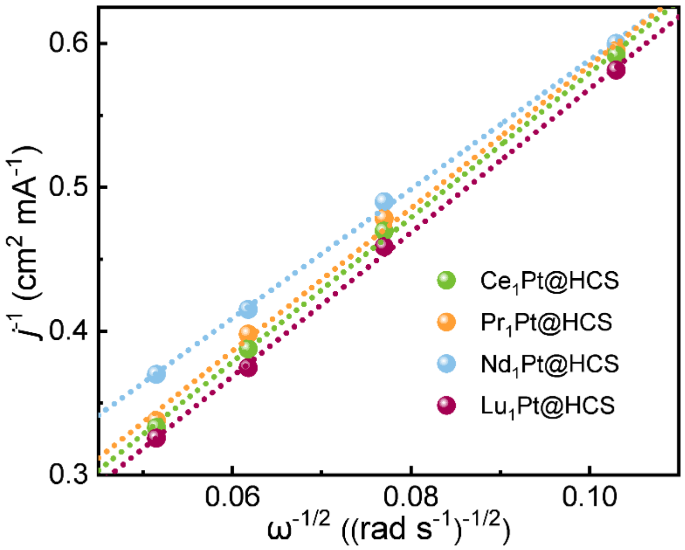

Supplementary Fig. 33 Electrocatalytic performance. The Koutechy-Levich plots of Ce_1Pt@HCS, Pr_1Pt@HCS, Nd_1Pt@HCS, and Lu_1Pt@HCS.

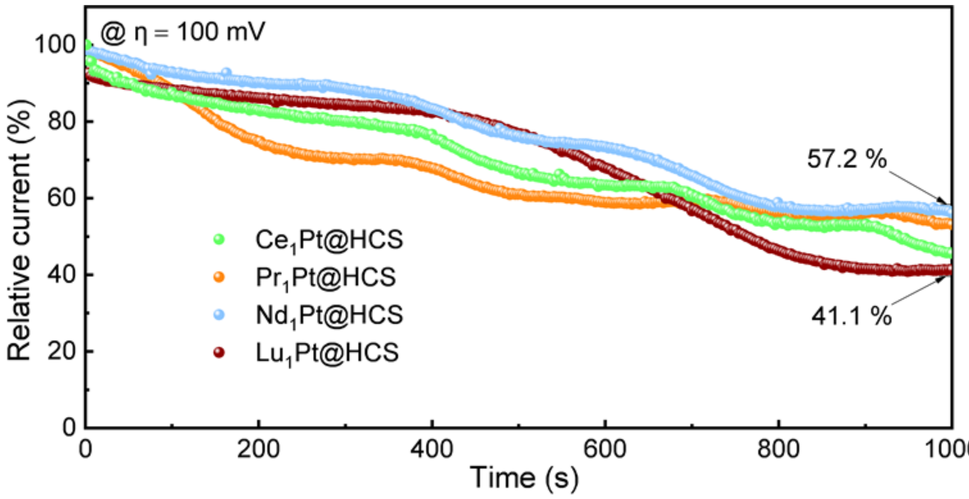

Supplementary Fig. 34 CO tolerance measurements. Chronoamperometry (*j*-*t*) response of other Ln_1Pt@HCS catalysts in CO (1000 ppm)/H_2-saturated 0.1 M KOH at 100 mV (vs. RHE).

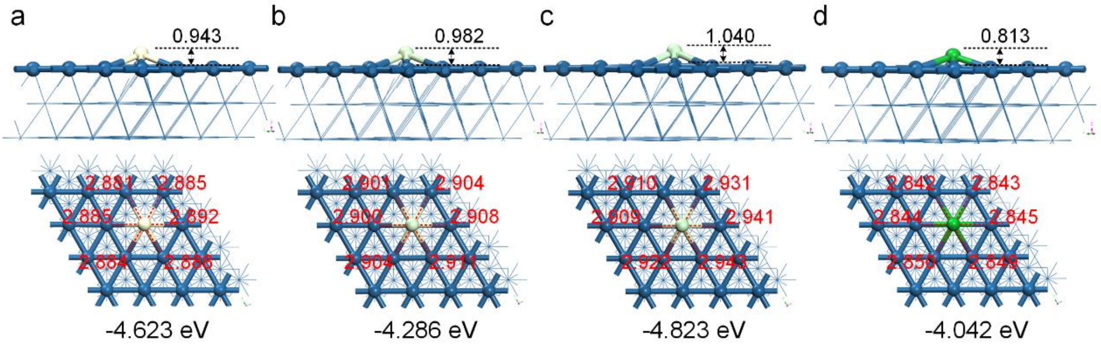

Supplementary Fig. 35 Optimization of other Ln_1Pt NCs models. a, Ce_1Pt; **b**, Pr_1Pt; **c**, Nd_1Pt; **d**, Lu_1Pt. Unit of bond length: Å. Pt, dark blue; Ln, another color. Bottom values represent formation energies for the Ln_1Pt system.

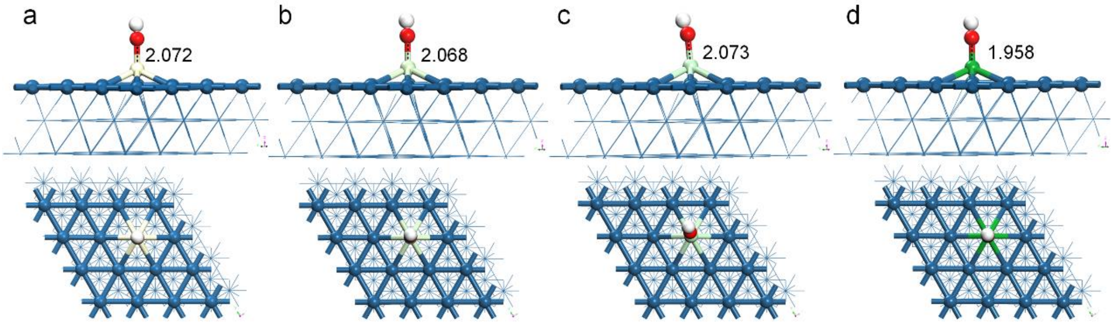

Supplementary Fig. 36 Model of OH adsorption on Ln_1 sites. a, Ce_1Pt; **b**, Pr_1Pt; **c**, Nd_1Pt; **d**, Lu_1Pt. Unit of bond length: Å.

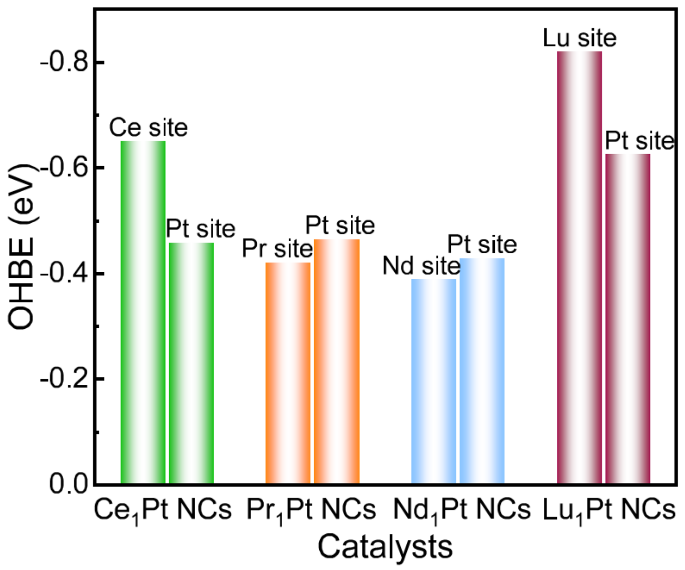

Supplementary Fig. 37 OHBE. Calculated OHBE on rare-earth sites and Pt sites for Ce_1Pt NCs, Pr_1Pt NCs, Nd_1Pt NCs, and Lu_1Pt NCs.

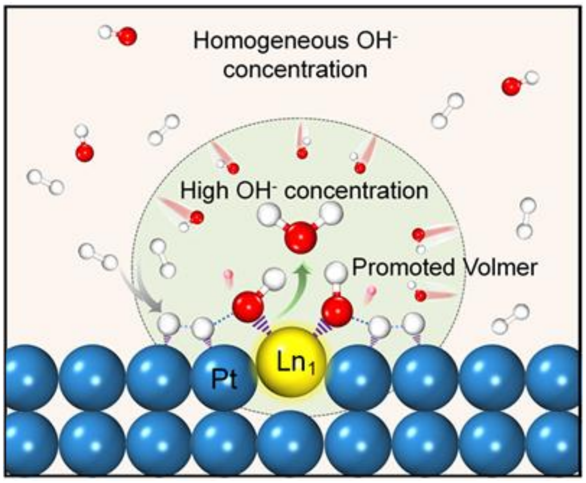

Supplementary Fig. 38 Mechanism model. Schematic illustration of the proposed HOR mechanism on Ln_1Pt NCs catalysts. Pt, dark blue; Rare-earth atom, golden; O, red; H, white.

Supplementary Tables

Supplementary Table 1 ICP-OES data of all Ln_1Pt@HCS electrocatalysts.

Element	La_1Pt@HCS	Ce_1Pt@HCS	Pr_1Pt@HCS	Nd_1Pt@HCS	Lu_1Pt@HCS
Ln (wt.%)	0.977%	0.845%	0.795%	0.941%	0.909%
Pt (wt.%)	8.598%	7.584%	8.785%	7.959%	8.091%

Supplementary Table 2 EXAFS fitting parameters extracted from the Pt L_3-edge for La_1Pt@HCS and reference samples.

Sample	Path	*CN*	*R* (Å)	σ^2(Å^2)	*R* factor
La_1Pt@HCS[a]	Pt-O	2.0 (0.6)	1.83 (0.2)	0.009	0.024
	Pt-O-O	4.1	2.64	0.005	
	Pt-Pt	5.1 (0.1)	3.05 (0.4)	0.003	
Pt foil	Pt-Pt	12.0	2.77	0.005	0.002
PtO_2	Pt-O	5.6	2.02	0.002	0.007
	Pt-Pt	7.0	3.10	0.003	
	Pt-Pt	3.5	3.84	0.001	

S_0^2: amplitude reduction factor; *CN*: coordination numbers; *R*: bond distance; σ^2: Debye-Waller factors; *R* factor: goodness of fit.

[a] S_0^2 was fixed as 1. ΔE_0 was refined as a global fit parameter, returning a value of (-5.3 ± 0.8) eV. Data range: $3.0 \le k \le 14.0$ Å^{-1}, $1.0 \le R \le 6.0$ Å.

Supplementary Table 3 EXAFS fitting parameters extracted from the La L_3-edge for La_1Pt@HCS and reference samples.

Sample	Path	*CN*	*R* (Å)	σ^2(Å^2)	*R* factor
La_1Pt@HCS[a]	La-O	2(0.5)	1.57(0.1)	0.003	0.015
	La-O-O	8	2.82	0.002	
La_2O_3	La-O	6.0	2.50	0.004	0.001

S_0^2: amplitude reduction factor; *CN*: coordination numbers; *R*: bond distance; σ^2: Debye-Waller factors; *R* factor: goodness of fit.

[a] S_0^2 was fixed as 0.95. ΔE_0 was refined as a global fit parameter, returning a value of (-6.2 ± 1.0) eV. Data range: $3.0 \le k \le 10.0$ Å^{-1}, $1.0 \le R \le 6.0$ Å.

Supplementary Table 4 Benchmark HOR activities and the relevant parameters of the PGM-based catalysts in alkaline electrolytes.

Catalysts	Catalyst loading [μg_{PGM} cm^{-2}_{disk}]	ECSA [m^2 g^{-1}_{PGM}]	$j_{0,s}$ [mA cm^{-2}_{PGM}]	Reference
La_1Pt@HCS	10	129.5	1.55	**This work**
Pt_6NCs/C	5	41.8	1.546	1
Pt_1SAs/C	5	14.8	0.149	
PtNPs/C	5	36.9	0.446	
Pt/C_{com}	10	32.2	0.351	
Pd_3Fe@Pt/C	1.87	46.9	0.511	2
Pd_3Co@Pt/C	1.87	43.9	0.570	
$PtNb/NbO_x$-C	–	45	0.8	3
Rh_2Sb NBs/C	6.4	1.14 (cm^2)	0.506	4
$Pt_{0.25}Ru_{0.75}$/N-C	50	117.3	1.41	5
Acid-PtNi/C	10	25.1	1.89	6
Pt/Cu NWs	86.02	35.9	2.1	7
$Pt_{0.8}Ru_{0.2}/C$	7.09	49.0	1.42	8
Pd/CB	14.4	3.2	0.038	9
Pd-CeO_2/CB		8.8	0.136	
Pd/OLC		7.8	0.043	
Pd-CeO_2/OLC		17.1	0.569	
Pt_7Ru_3 NW	10	0.176 (cm^2)	0.493	10
PtRh	25.5	32.1	1.25	11
$PtRu/Mo_2C$	13	–	0.17	12
$PtRu/Mo_2C$-TaC	13	–	0.28	
$PtRu/Mo_2C$-W_2C	13	–	0.19	

Supplementary Table 5 Comparison between the La_1Pt and other catalysts in HOR activities including Tafel reaction barrier (H_2-DE), Volmer reaction barrier (H_2O formation), HBE, OHBE, and H_2O binding energies (H_2OBE)[a].

Systems	H_2-DE	H_2O formation	HBE	OHBE	H_2OBE	Reference
La_1Pt	0.27	0.42	-0.44	-0.35	-1.43	**This work**
Ni(111)	0.20	0.94	-0.72	-1.14	–	13
PtNi(111)	0.15	0.93	-0.58	-0.25	–	13
Pt(110)	0	0.41	-0.62	-3.51	-0.47	14
CeO_2	1.82	0.69	-1.34	-1.32	-0.53	15
Pd(111)	0.03	0.98	-0.69	-3.33	-0.42	15
Pd@CeO_2	0.05	0.48	-1.29	-3.25	-0.89	15

[a] All energies are in eV units. The values are rounded up to two decimal digits.

Supplementary References

1. Wang X*, et al.* Atomic-precision Pt_6 nanoclusters for enhanced hydrogen electro-oxidation. *Nat. Commun.* **13**, 1596 (2022).
2. Zhao T*, et al.* Electronic structure and oxophilicity optimization of mono-layer Pt for efficient electrocatalysis. *Nano Energy* **74**, 104877 (2020).
3. Ghoshal S*, et al.* Tuning Nb-Pt interactions to facilitate fuel cell electrocatalysis. *ACS Catal.* **7**, 4936-4946 (2017).
4. Zhang Y*, et al.* Atomically isolated Rh sites within highly branched Rh_2Sb nanostructures enhance bifunctional hydrogen electrocatalysis. *Adv. Mater.* **33**, 2105049 (2021).
5. Cong Y, Chai C, Zhao X, Yi B, Song Y. $Pt_{0.25}Ru_{0.75}$/N-C as highly active and durable electrocatalysts toward alkaline hydrogen oxidation reaction. *Adv. Mater. Interfaces* **7**, 2000310 (2020).
6. Lu S, Zhuang Z. Investigating the influences of the adsorbed species on catalytic activity for hydrogen oxidation reaction in alkaline electrolyte. *J. Am. Chem. Soc.* **139**, 5156-5163 (2017).
7. Alia SM, Pivovar BS, Yan Y. Platinum-coated copper nanowires with high activity for hydrogen oxidation reaction in base. *J. Am. Chem. Soc.* **135**, 13473-13478 (2013).
8. St. John S, Atkinson RW, Unocic RR, Zawodzinski TA, Papandrew AB. Ruthenium-alloy electrocatalysts with tunable hydrogen oxidation kinetics in alkaline electrolyte. *J. Phys. Chem. C* **119**, 13481-13487 (2015).
9. Ogada JJ, *et al*. CeO_2 modulates the electronic states of a palladium onion-like carbon interface into a highly active and durable electrocatalyst for hydrogen oxidation in anion-exchange-membrane fuel cells. *ACS Catal.* **12**, 7014-7029 (2022).
10. Scofield ME, *et al*. Role of chemical composition in the enhanced catalytic activity of pt-based alloyed ultrathin nanowires for the hydrogen oxidation reaction under alkaline conditions. *ACS Catal.* **6**, 3895-3908 (2016).
11. Jin Y, Chen F, Wang J, Guo L, Jin T, Liu H. Lamellar platinum-rhodium aerogels with superior electrocatalytic performance for both hydrogen oxidation and evolution reaction in alkaline environment. *J. Power Sources* **435**, 226798 (2019).
12. Hamo ER*, et al.* Carbide-supported PtRu catalysts for hydrogen oxidation reaction in alkaline electrolyte. *ACS Catal.* **11**, 932-947 (2021).
13. García-Melchor M, López N. Homolytic products from heterolytic paths in H_2 dissociation on metal oxides: The example of CeO_2. *J. Phys. Chem. C* **118**, 10921-10926 (2014).
14. Feng Z*, et al.* Role of hydroxyl species in hydrogen oxidation reaction: A DFT Study. *J. Phys. Chem. C* **123**, 23931-23939 (2019).
15. Sahoo S, Dekel DR, Maric R, Alpay SP. Atomistic Insights into the hydrogen oxidation reaction of palladium-ceria bifunctional catalysts for anion-exchange membrane fuel cells. *ACS Catal.* **11**, 2561-2571 (2021).
